# Experimental Evaluation of the Concrete Damage and Pore Characteristics under Salt-Freezing Cycles

**DOI:** 10.3390/ma15134454

**Published:** 2022-06-24

**Authors:** Jiguo Zhou, Guihua Wang, Jun Xu

**Affiliations:** 1 College of Civil Engineering, Baicheng Normal University, Baicheng 137000, China; wangguihua@bcnu.edu.cn; 2 College of Civil Engineering and Architecture, Jiangsu University of Science and Technology, Zhenjiang 212003, China; xujun@just.edu.cn

**Keywords:** salt freezing effect, concrete durability, compressive strength, DEM, pore characteristics

## Abstract

Herein, ordinary silicate concrete specimens are prepared to study the damage law of a cement-concrete material under the effects of salt erosion and a freeze–thaw environment. NaCl, NaHCO_3_, and Na_2_SO_4_ solutions are separately produced, according to the characteristics of saline soil, to conduct an experimental study on the concrete characteristics during quick salt freezing cycles, and to analyse the changes in its compressive strength, mass loss, and dynamic elastic modulus (DEM) under freeze–thaw cycles. Low-field nuclear magnetic resonance (NMR) and scanning electronic microscopy are used to investigate the change in the microstructure of concrete specimens under salt freeze–thaw cycles (FTCs). The results show the loss in compressive strength, mass, DEM, and NMR spectrum signal increased by 1.5–3 times, 3–5 times, 1.5–2.5 times, and 2–4 times, respectively, for concrete specimens under 50–100 FTCs in 6.8% composite salt solution, in comparison to fresh water. Apparent spalling, decreases in the DEM, and reductions in the compressive strength occur in concrete when increasing the number of salt FTCs. The number of internal cracks in the concrete structure increase under the combined action of salt crystallization, moisture absorption, and freeze–thaw. The changes in the internal microscopic pore volume in concrete structures exhibit the same trend with changes in the macro mechanical properties of concrete. The correlation coefficients between the changes in each peak in the NUR spectrum and the changes in the compressive strength of concrete specimens under FTCs in freshwater or low-concentration salt solutions are both larger than 0.7, calculated using the grey correlation degree method. Therefore, these changes could be used as a potential evaluation index for salt frozen damage to concrete structures.

## 1. Introduction

Freeze–thaw action significantly affects the durability of concrete; this action more severely damages the macroscopic properties of concrete when the environment experiences freezing and thawing over a long period [1]. Research has shown that FTCs lead to surface erosion in concrete and significantly decrease its compressive strength [2]. The action of FTCs has an obvious influence on the safety and durability of concrete structures; therefore, investigating the frost resistance and the damage law of concrete materials under FTCs can help understand the service life of concrete structures and construction elements [3]. FTCs in a salt environment will accelerate the freeze–thaw damage of concrete materials in comparison to those in a freshwater environment [4]. The apparent morphology, dynamic modulus, compressive strength, and microstructure of concrete under FTCs exhibit regular variations [5]. The change in the microstructural and macroscopic properties of concrete materials present a comparable varying regularity when experiencing damage under FTCs [6]. Therefore, it is of great significance to understand the change in the DEM of concrete structures, in addition to the distribution characteristics of microscopic pores, when studying the macroscopic mechanical properties of concrete [7].

Salt environments significantly affect the durability of concrete structures, as salt erosion reduces their service life [8]. The combined effect of FTCs and salt intrusion will accelerate the deterioration of concrete structure durability [9], and cracks will continuously be generated during the freeze–thaw process [10].

Li [6] carried out research to study the physical and mechanical properties, microstructural features, mechanism of chloride ion transport, and damage fracture mechanism of concrete mixed with admixtures under marine corrosion and a freeze–thaw environment. Yao et al. [11] studied the performance degradation processes of concrete specimens within 60 salt FTCs, including changes in the apparent phenomenon, flaking amounts, and compressive strength loss. According to their study, the internal concrete structure becomes loose and flocculent, an increasing number of pores and cracks appear, and the density decreases, which results in a deterioration of the macro-mechanical properties. Zheng et al. [12] carried out concrete frost-resistance durability tests and used ICT scanning technology to study the two-dimensional and three-dimensional ICT pore characteristics of concrete specimens before and after freezing and thawing in different solutions. According to their results, with increases in the freezing and thawing times, the concrete porosity, pore area, pore volume, and pore number generally increased in any solution environment inside the concrete structure, resulting in losses in the concrete compressive strength, mortar spalling, and decreases in the relative DEM. Xiang et al. [13] measured the mass loss, strength deterioration, ultrasonic longitudinal wave velocity, and DEM, establishing a prediction model for freeze–thaw damage to explore the occurrence area of pervious concrete freeze–thaw deterioration. Their results show that the damage sensitivity and strength loss of freeze–thaw damage is greater than the DEM loss, which is greater than the mass loss. However, in pervious concrete paste that underwent 100 freeze–thaw cycles, the pore structure and macro strength exhibited no obvious changes, and the hardened paste and the aggregate–interface-generated defects increased with increases in the freezing and thawing time.

The mechanical properties of concrete structures, such as concrete dams and bridges, are greatly affected by freeze–thaw cycles [14,15,16]. Scholars worldwide have carried out systematic studies on the durability damage mechanism, degradation law, and durability repair and promotion, obtaining many meaningful results, including single and multi-factor coupling actions and research on materials and structures [17,18,19]. In previous studies, few attempts have been made to study the coupled properties of micro-structure changes, chloride ion penetration, and mechanical properties of cement concrete. Further research is needed to study the relationship between chloride diffusion, mechanical properties, and the microstructure of concrete structures. Research combining the macro mechanical properties with pore changes inside the concrete structure to ascertain the mechanism of load influences is crucial [20]. Although many scholars have carried out relevant studies on the physical properties and ion transport properties for concrete under salt freeze environments, the results diverge significantly [21], and there are discrepancies between test methods and theoretical models. It is necessary to characterize the damage process of concrete through internal pore structure evolution, to establish more realistic damage variability combined with concrete material ratios and specific environmental conditions. The soil environment in Northeast China is more saline, which leads to salt frost damage in concrete structures. This experiment takes the saline soil environment in western Jilin as the engineering background [22], and studies the change law of macroscopic and microcosmic performance deterioration of cement concrete under FTCs in a composite salt solution and single salt solution of compound salt, in addition to its mechanical properties. Ultrasonic non-destructive testing is applied to ascertain the change law of the DEM of concrete specimens. Low-field NMR technology, scanning electron microscopy, and X-ray diffraction phase analysis are used to observe changes in the microscopic pore structure and the composition characteristics of concrete materials. The damage properties of cement concrete under salt FTCs are studied according to the macroscopic and microscopic performance changes of concrete under these cycles.

## 2. Materials and Methods

### 2.1. Test Materials

Ordinary Portland cement 42.5 is used as the cement mortar material; its main parameters are listed in Table 1. The fine aggregates of cement concrete are medium sand, with a fineness modulus of 2.3 and an apparent density of 2646 kg/m^3^. The coarse aggregates of cement concrete are graded crushed stone, with a particle size of 2–52 mm. The concrete mix proportion design is based on the code ‘specification for mix design of common concrete’ [23]. The proportions of components in cement concrete are listed in Table 2. The concrete material studied in this paper is based on that used in the durability study on concrete building structure without air-entraining agents in the saline soil of western Jilin, China. The air-entraining agent is not reflected in the concrete ratio, and the air content of the concrete material is 2%. The specimens are conserved for the 28-day standard, according to the standard test method for the physical and mechanical properties of cement concrete [24]; the average compressive strength of the cube specimen is 42.5 MPa. Where the compressive strength of cement and concrete blocks were decided following Chinese Standard GB/T 50081-2019 and the Standard for Test Methods of Concrete Physical and Mechanical Properties; 7-day and 28-day denote the number of days the cement and concrete specimens were maintained in a standard curing room.

### 2.2. Test Method

Cubic specimens with dimensions of 100 mm × 100 mm × 100 mm are fabricated to test the compressive strength of concrete. Further, cuboid specimens with dimensions of 100 mm × 100 mm × 400 mm are made to test the DEM of concrete.

The tested concrete blocks were fabricated following Chinese Standard GB/T 50081-2019 [24]. The cement concrete specimens were kept in a standard curing room with a humidity of no less than 95% and a temperature of 20 ± 2 °C after pouring operations. The experiments began by carrying out rapid FTCs on the cement concrete specimens when the curing age was 28 days. The rapid freeze–thaw tests of concrete are carried out based on the Chinese Standard GB/T 50082-2009 [25]. The compressive strength, mass, and DEM loss of concrete are measured when the quick FTCs are 0, 25, 50, 75, and 100. The solution in each test box uses freshwater, composite salt, sodium chloride, sodium bicarbonate, and sodium sulfate solution. Ordinary tap water is used as freshwater. The ratio of the salt solution of single salt and compound salt for the rapid FTC experiment was prepared according to the contents of Na^+^, SO_4_^2−^, Cl^−^, and HCO_3_^−^ in soil layers 30 cm away from the surface in western Jilin, China [22]. Each single salt solution was twice the proportion of each salt in the saline soil. In each prepared single salt solution, the mass of Na_2_SO_4_ was 26.72 g/L, the mass of NaCl was 14.92 g/L, and the mass of NaHCO_3_ was 28.76 g/L. The amount of compound salt was the sum of each individual salt content.

The WAW-600 press machine is used to conduct the compressive strength experiment, the DT-20 ammunition instrument is used to test the DEM, MRE-60 low-field NMR is used to measure the pore characteristics of the material, the HC-RCTF quick tester for chloride ion contents is used to analyse the chloride concentration in each layer of the concrete material under the freeze–thaw action in a NaCl solution, the SU8010 scanning electron microscope is used to analyse the microstructural characteristics of the material, and the X’Pert^3^ Powder X-ray powder diffraction machine is used to ascertain the composition of the material every 25 FTCs.

The concrete specimens were analysed by XRD using X’Pert III equipment (Malvern Panalytical Ltd., Eindhoven, The Netherlands). The CuKα radiation was recorded in the 2θ range of 10°and 70°, using a single-channel detector. The radiation was collected at a step size of 0.013°, while the copper X-ray tube was operated at a 40 mA current intensity and a 40 kV voltage. The XRD analysis was performed on samples in a powder form, which were ground after the curing period. The NMR wave test was conducted using the MesoMR12-060H equipment (Suzhou NUMAG Analytical Instrument Corporation, Suzhou, China), and the magnetic field intensity of the instrument was 0.3 ± 0.05 T (Tesla). The CPMG pulse sequence was applied to characterize the lateral relaxation time spectra (*T*_2_) of the pore size of the concrete structure, the sampling frequency was 250 kHz, the echo interval was 0.1 ms, the echo number was 12,000, and the signal ratio was greater than 100.

## 3. Mechanical Performance of Concrete

### 3.1. Compressive Strength Change

**Figure 1:**
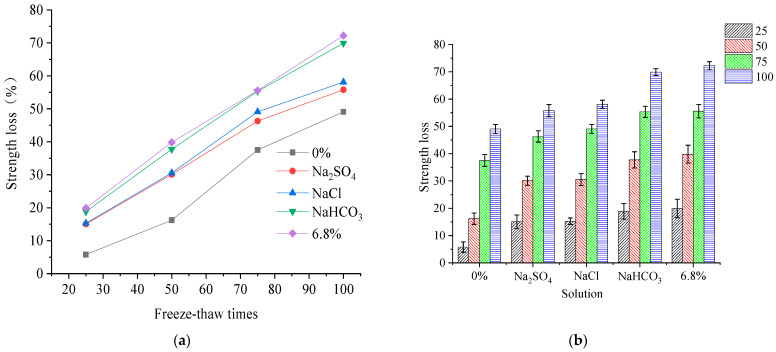
(**a**) loss of compressive strength of concrete under FTCs in different salt solutions; (**b**) loss of compressive strength of concrete under different freeze–thaw times.

The change law of compressive strength is tested to understand the mechanical damage to concrete under quick FTCs in different solutions. The compressive strength loss of concrete under the freezing and thawing action in freshwater, sodium chloride, sodium bicarbonate, sodium sulfate, and 6.8% composite salt solution are shown in Figure 1. Three concrete block samples were measured for compressive strength tests each time, and the statistics followed the Chinese Standard GB/T 50081-2019 [24].

The compressive strength loss of each specimen increases with the number of FTCs, and has a linear relation in the freshwater, three single salt, and composite salt solutions. When the number of FTCs is 100, the loss in compressive strength of concrete specimens under rapid FTCs in freshwater is 3 times that of 50 rapid FTCs. When the number of FTCs is 100, the loss of compressive strength of concrete specimens under rapid FTCs in single and compound salt solutions is twice that of 50 rapid FTCs. Comparing the compressive strength loss in different solutions, the compressive strength loss is at a minimum when the concrete experiences freshwater FTCs, and the compressive strength loss is at a maximum when the concrete experiences composite salt solution FTCs. When the number of FTCs is 50–100, the loss in compressive strength of concrete specimens under FTCs in 6.8% composite salt solution is 1.5–3.0 times that of FTCs in fresh water. The salt concentration of three single salt solutions prepared according to the characteristics of saline soil in western Jilin, China, is non-conforming. Comparing the compressive strength loss of concrete specimens under FTCs in the three single salt solutions, it can be seen that there are differences in the effects on the concrete owing to variations in the concentration of a single salt solution. Therefore, comparing the compressive strength loss with the salt solution concentration shows that the compressive strength loss is maximum when the concrete specimen is in a NaCl solution, and the compressive strength loss rate is similar when concrete specimens are in Na_2_SO_4_ and NaHCO_3_ solutions.

### 3.2. Dynamic Elastic Modulus Change

**Figure 2:**
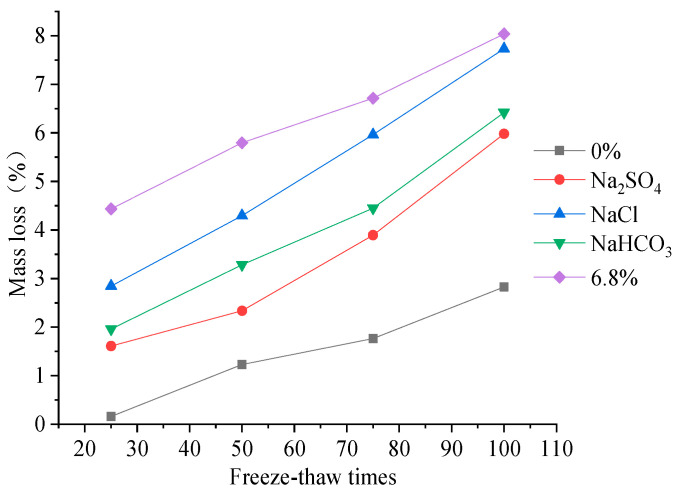
Loss of mass of concrete.

**Figure 3:**
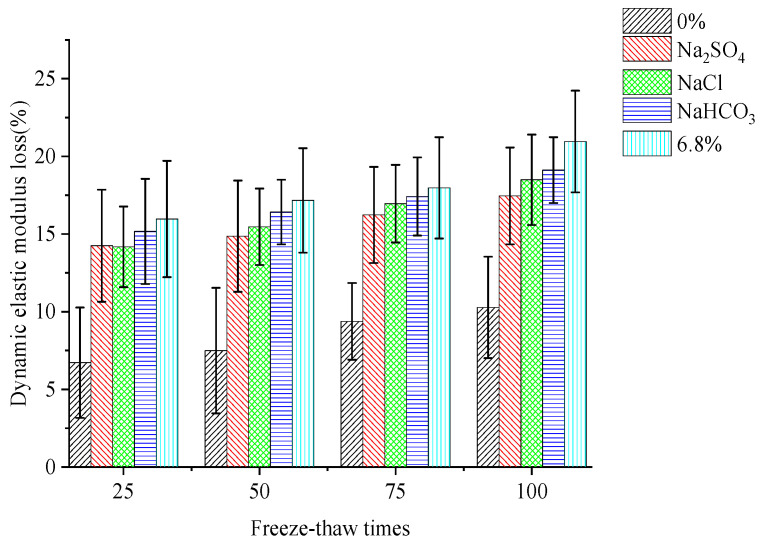
Loss of DEM of concrete.

Studies have shown that changes in the DEM of concrete can reflect the internal damage to the concrete specimen under FTCs, which indicates the change in the compressive strength loss of concrete [26]. The changes in the mass and DEM of concrete specimens under FTCs in the five different solutions were tested to compare the variation characteristics of the compressive strength and DEM of concrete. Three concrete block samples were measured for the DEM test each time, and the statistics followed Chinese Standard GB/T 50082-2009 [25]. The loss rule of the mass and DEM of concrete under fast freeze–thaw action are shown in Figure 2 and Figure 3, respectively.

The mass loss of concrete specimens under freeze–thaw action in freshwater and salt solutions increases with increases in the FTCs. There is an approximately linearly proportional relationship between the mass loss of concrete and the freeze–thaw cycle number. When the number of FTCs is 100, the loss in the mass of concrete specimens under rapid FTCs in freshwater is 2.5 times that of 50 rapid FTCs. When the number of FTCs is 100, the loss of mass of concrete specimens under rapid FTCs in single and compound salt solutions is 1.5–2.5 times that of 50 rapid FTCs. By comparing the variation characteristics of concrete properties under the action of different solutions, it can be seen that the mass loss of concrete is the smallest under the action of freshwater, and the mass loss is the largest under the freeze–thaw action in the composite salt solution. When the number of FTCs is 50–100, the loss in mass of concrete specimens under FTCs in a 6.8% composite salt solution is 3.0–5.0 times that of FTCs in fresh water. Comparing the action of different single salt solutions, the mass loss of concrete is the largest under freeze–thaw action in the NaCl solution, and the mass loss is relatively smaller under freeze–thaw action in the Na_2_SO_4_ solution. The results show that the NaCl salt solution freeze–thaw action leads to the most severe surface erosion of concrete.

The DEM loss of concrete specimens in different solutions increase with increases in the number of freeze-thaw cycles. When the number of FTCs is 100, the loss in DEM of concrete specimens under rapid FTCs in freshwater or salt solutions is 1.2–1.5 times that of 50 rapid FTCs. When the number of FTCs is 50–100, the loss in DEM of concrete specimens under FTCs in a 6.8% composite salt solution is 1.5–2.5 times that of FTCs in fresh water. It can be seen that the DEM loss rate is larger than the mass loss rate of concrete from the characteristics changes of concrete under different numbers of FTCs. The DEM damage of concrete under freeze–thaw action in freshwater is smaller than that in salt solutions. The DEM loss of concrete under freeze–thaw action in composite solutions is larger than that in single salt solutions. Comparing the DEM loss of concrete under FTCs in three different single salt solutions shows that the DEM loss is largest in a NaHCO_3_ solution, and the DEM loss is relatively smaller in NaCl and Na_2_SO_4_ solutions.

By comparing the compressive strength loss, mass loss, and DEM loss of concrete under rapid freeze–thaw action in different solutions, it can be seen that there is an approximately linear relationship between the three indexes of loss change of concrete and the number of FTCs. Comparing the three damage indexes of concrete under freeze–thaw action in different solutions, the change characteristics of compressive strength loss are consistent with those of the dynamic modulus loss of concrete. By comparatively analysing the three single salt solutions, it can be seen that the effect of NaHCO_3_ on the compressive strength and DEM of concrete is more severe in the saline soil environment of western Jilin, China, and the effect of FTCs in NaCl solutions on the mass loss of concrete shows that the surface erosion of concrete is more serious. Comparing the freeze–thaw cycle action in the composite salt solution, single salt solution, and freshwater, it can be seen that the damage to the internal structure caused by the FTCs of composite salt solutions is more severe than that caused by the single salt solution and freshwater. This also shows that the combined action of saline oil and the seasonal frozen environment in western Jilin, China, will lead to more severe damage to cement concrete structures.

## 4. Erosion Effects of Chloride Ions on Concrete

**Figure 4:**
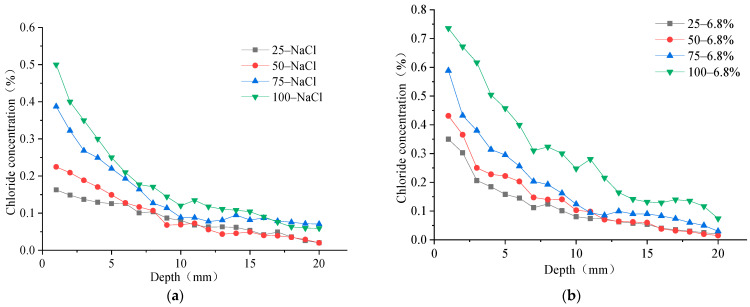
Chloride ion concentration in concrete under freeze–thaw action in a (**a**) NaCl solution and (**b**) composite salt solution.

One surface of the concrete specimen was used as the chloride ion erosion surface, and the other five surfaces were smeared with epoxy resin to study the erosion law of chloride ions in the concrete specimen under freeze–thaw action in the salt solution. The concrete specimens were put into the NaCl solution and composite salt solution test box for rapid freeze–thaw cycle testing, and the mass percentage of chloride ions at different depths in the concrete specimen under the freeze–thaw action of two different salt solutions were tested at the 25, 50, 75, and 100 freeze–thaw times. The diffusion rule of chloride ions in concrete is shown in Figure 4.

**Figure 5:**
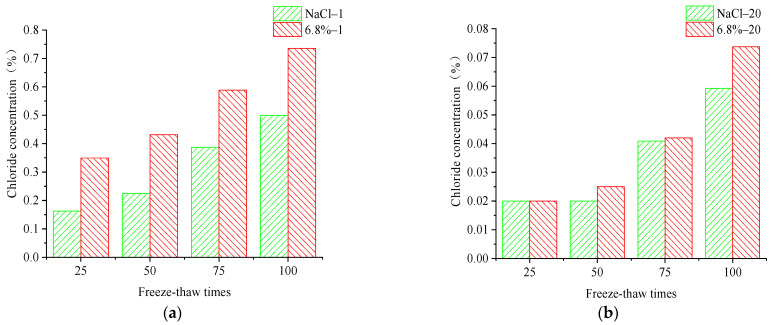
Chloride ion concentration at (**a**) 1 mm from the erosion surface and (**b**) 20 mm from the erosion surface.

It can be seen that the chloride ion intrusion concentration decreases rapidly at 0 mm–10 mm away from the erosion surface of concrete under different numbers of FTCs, and subsequently decreases slowly at 10–20 mm away from the surface. The overall chloride ion concentration varies exponentially with erosion depth. This proves that the chloride ions rapidly erode the concrete surface, and the erosion speed decreases with erosion depth away from the concrete surface. The chloride ion concentration increases with the number of FTCs at the surface up to the 20 mm position from the erosion surface. Further, it can be seen that the chloride diffusion speed in the concrete structure under the freeze–thaw action in the composite salt solution is higher than that in the NaCl solution. The changes of chloride ion concentration with FTCs number at 1 mm and 20 mm from the erosion surface are shown in Figure 5.

By comparing the change rule of chloride ion concentration at the 1 mm and 20 mm erosion position of concrete under the FTCs in two salt solutions, it can be seen that the chloride ion concentration is greater at the different erosion positions of the concrete specimen under the FTCs in the composite salt solution; in other words, the chloride ion penetration ability is higher in the composite salt solution than in the NaCl solution. By comparing the chloride ion concentration at different depths, it can be seen that the chloride ion centration steadily increases with the number of FTCs at a depth of 0–10 mm. At the depth of 10–20 mm away from the erosion surface, the chloride ion concentration grows slowly in the concrete at 0–50 times of freeze–thaw cycle action. The chloride ion concentration grows faster at the depth of 10–20 mm away from the erosion surface when the number of FTCs increases to 75–100 times. That also proves that the chloride ion erosion is more serious at the concrete surface under freeze–thaw action in the salt solution. The chloride ions erode significantly into the interior of the concrete specimen with increases in the number of FTCs, which is consistent with the law of apparent erosion of concrete under the action of freeze–thaw.

## 5. Pore Structure Characteristics of Concrete

**Figure 6:**
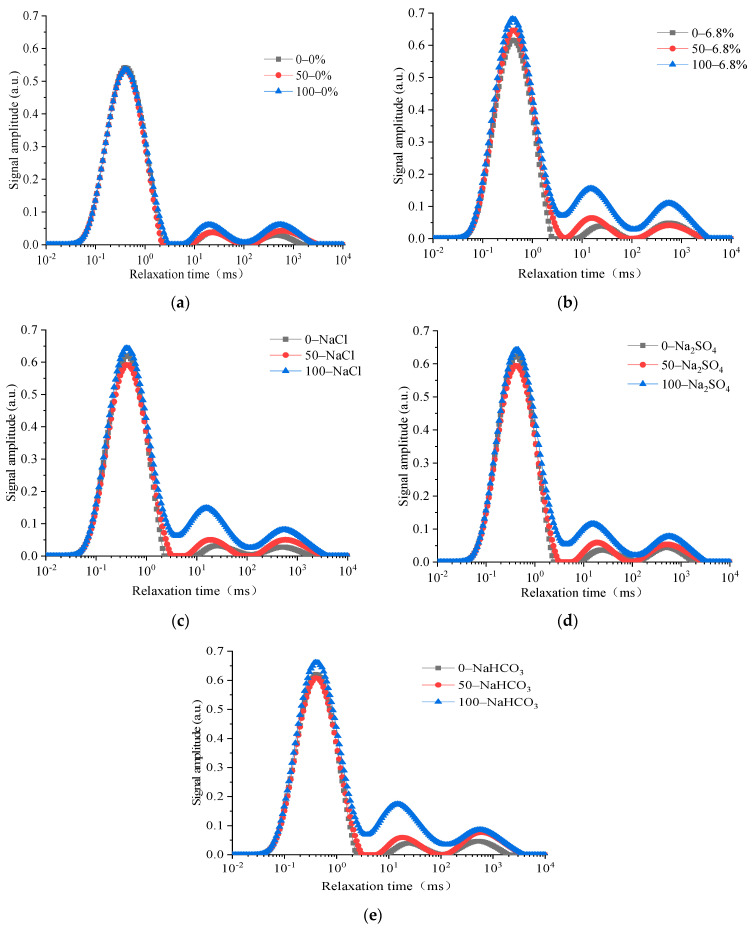
Nuclear magnetic *T*_2_ spectrum of concrete under freeze-thaw cycle action in (**a**) clean water; (**b**) a complex salt solution; (**c**) a NaCl solution; (**d**) a Na_2_SO_4_ solution; and (**e**) a NaHCO_3_ solution.

**Figure 7:**
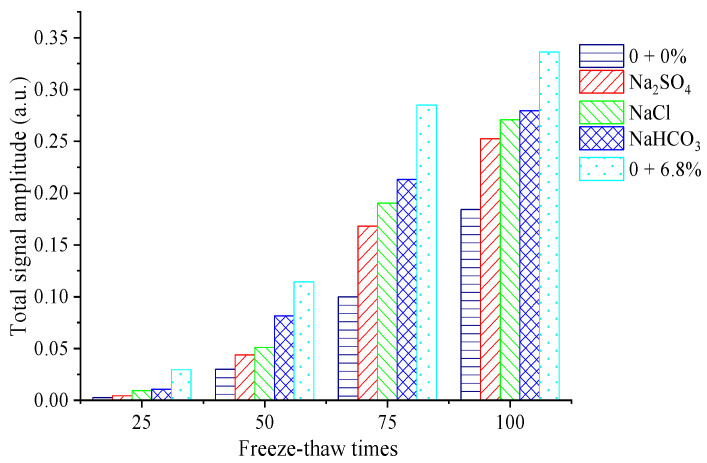
Area of the nuclear magnetic *T*_2_ spectrum.

Low-field NMR technology, as an effective non-destructive testing method, has been applied to the microscopic pore structure detection of cement-based materials [27]. The pore size distribution and volume ratio measured by NMR technology can be used as an indicator to reflect the properties of concrete under freeze–thaw action [28]. Based on the NMR mechanism, the characteristics of the transverse relaxation time *T*_2_ of water-saturated porous media can be measured by CPMG sequencing, and the pore distribution characteristics in the concrete can be obtained using the surface relaxation rate [29]. According to the CPMG pulse echo sequence, the pore proportion characteristics of each concrete specimen could be obtained through the numerical solution using the inverse Laplace inversion algorithm [30]. Comparing the pore structure variation characteristics of concrete under the FTCs in different salt solutions, the *T*_2_ spectrum characteristics of each specimen are shown in Figure 6 when the number of FTCs is 0, 50, and 100 times. The change in the unit mass area of the *T*_2_ spectrum is shown in Figure 7.

The transverse coordinate in the *T*_2_ spectrum represents the relaxation time and is shown in logarithmic coordinates. The relaxation time of the *T*_2_ spectrum corresponded to special pore diameters [31]: micropores and transition pores (<2.5 ms), middle-sized pores (2.5–100 ms), and large pores and cracks (>100 ms). When NMR relaxation tests were performed on porous materials filled with water or other hydrogen-rich liquids, the contribution of free relaxation and diffusion relaxation can be ignored under the conditions of strict control of magnetic resonance instrument and specific testing parameters. At this time, the lateral relaxation rate of liquid is determined by the specific surface area of the porous materials. The lateral relaxation time *T*_2_ is approximately linearly related to the equivalent radius of the pores, *r* = 2*ρ*_2_*T*_2_ [30]. Here, *ρ*_2_ is the surface relaxation rate. The pore sizes of the concrete specimens measured through NMR in this paper are mainly distributed in the range of 1 nm–100 μm, when *ρ*_2_ is 10 μm/s. The longer the relaxation time in the transverse coordinates, the greater the pore diameter. The ordinates in the *T*_2_ spectrum represent the signal intensity of the NMR inversion of the pores, and the value of the signal intensity characterizes the ratio of the pore diameter in the corresponding transverse coordinate. The larger the ordinate, the larger the proportion of the correspondingly sized pores in the entire material.

It could be seen that the *T*_2_ spectrum of each concrete specimen exhibits three peaks. The size of each peak increases with the number of FTCs, indicating that the pore proportions in the concrete specimen change. The transverse relaxation time corresponding to each peak of the *T*_2_ spectrum, which represents the pore size distribution, remains basically unchanged with the increasing number of FTCs. Therefore, the properties of the *T*_2_ spectrum indicate that there are three main types of pore structure in the internal structure of concrete under the FTCs. The intensity change of each peak of the *T*_2_ spectrum of the concrete specimen is relatively small under changing FTCs in freshwater than in composite salt solutions. Salt gradually penetrates into the concrete under FTCs in a salt solution. The signal intensity of the *T*_2_ spectrum of each concrete specimen increases overall, but the characteristics of pore growth are different under the combined action of salt crystallization and freezing and thawing. The first peak of the *T*_2_ spectrum of the concrete specimen under the freeze–thaw cycle in the salt solution is relatively large, which indicates that the proportion of micropores in the structure is large. The area of the first peak of the *T*_2_ spectrum changes slowly with the increasing number of FTCs. However, the area of the second and third peaks of the *T*_2_ spectrum increases significantly with an increasing number of FTCs, indicating that the change in the mesopores and macropores of concrete specimens under freeze–thaw action in a salt solution is more sensitive than that for micropores.

Variations in the unit mass *T*_2_ spectrum can reflect the distribution characteristics of the pore water volume of unit mass solid skeletons in the concrete structure [29]. The total signal intensity of the *T*_2_ spectrum of the unit mass of each specimen increases with increases in the number of FTCs, which shows that the internal cracks and pore proportion both increase with freeze–thaw action. When the number of FTCs is 100, the area of the *T*_2_ spectrum per unit mass of concrete specimens under rapid FTCs in freshwater is 6.0 times that of 50 rapid FTCs. When the number of FTCs is 100, the area of the *T*_2_ spectrum per unit mass of concrete specimens under rapid FTCs in single and compound salt solutions is 3.0–6.0 times that of 50 rapid FTCs. The changes in the pore within the concrete are caused by freeze–thaw damage. Therefore, the variation characteristics of the *T*_2_ spectrum indicate the variation rule of pore characteristics in concrete, and the corresponding macroscopic phenomenon is the decrease in the compressive strength and DEM for the concrete under FTCs.

Comparing the signal change of the unit mass *T*_2_ spectrum of each specimen under FTCs in a single salt solution, it can be seen that the total signal of the *T*_2_ spectrum per unit mass of concrete under freeze–thaw action in the NaHCO_3_ solution is the largest, which indicates that the crack growth rate of concrete specimens under NaHCO_3_ salt freezing is the fastest, and the damage to the concrete specimen caused by the freeze–thaw cycle is most obvious.

The total signal of the *T*_2_ spectrum per unit mass under the freeze–thaw cycle in freshwater is smaller than that in three single salt solutions, which shows that the damage to the concrete specimen caused by freeze–thaw action in a composite salt solution is more serious than that in freshwater. When the number of FTCs is 50–100, the area of the *T*_2_ spectrum per unit mass of concrete specimens under FTCs in a 6.8% composite salt solution is 2.0–4.0 times that of FTCs in fresh water. It can be seen from the comparison of the total signal intensity of the *T*_2_ spectrum per unit mass of concrete under the action of FTCs in a composite salt solution and three single salt solution that the pore growth speed of concrete in the composite salt solution is faster than that in a single salt solution. This shows that the crystallization pressure and hygroscopicity generated by the three single salts in the composite salt increase the freezing pressure in the concrete, leading to more severe damage to the concrete and more cracking under freeze–thaw action in a composite salt solution. This also explains why the chloride ion concentration at different depths in concrete under freeze–thaw action in a composite salt solution is higher than that in the single NaCl solution.

## 6. Component Characteristics of Concrete

### 6.1. Principle Component Analysis

**Figure 8:**
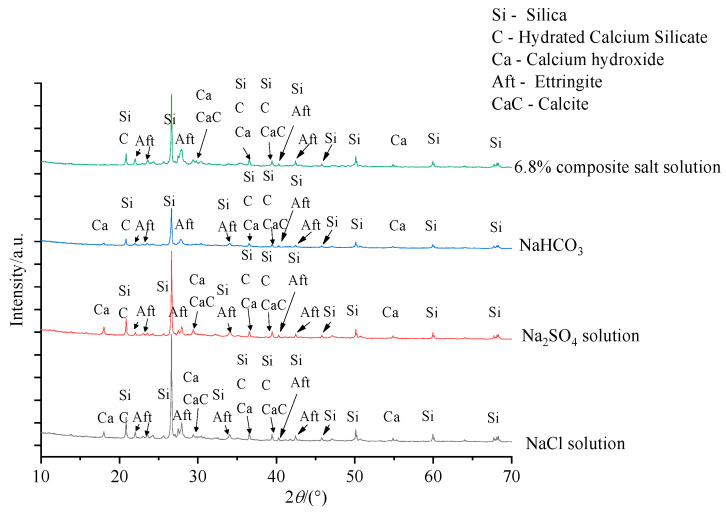
XRD spectrum of concrete.

To compare and analyse the composition characteristics of concrete material under FTCs in each salt solution, the concrete specimens were frozen and thawed 100 times in three types of salt and composite salt solutions. The X-ray powder diffractometer was used to observe the main components of concrete materials at the 2-mm position away from the erosion surface. The XRD spectrum obtained is shown in Figure 8.

From the XRD spectrum, it can be seen that the main components of the concrete material are silica (SiO_2_), gel (C-S-H), calcium hydroxide (Ca(OH)_2_), and calcium sulphoaluminate hydrate (Aft/AFm). Each kind of salt gradually penetrates into the interior concrete and crystallizes to produce a larger crystal pressure with an increasing number of FTCs. There will be a greater ice pressure in concrete under the negative effect of salt, which will lead to more cracks being generated in the concrete’s internal structure, which leads to changes in the signal of the *T*_2_ spectrum and macroscopic mechanical properties of concrete. The intrusion of NaCl into the concrete leads to a loss of Ca(OH)_2_; the reaction of Na_2_SO_4_ and Ca(OH)_2_ will generate (CaSO_4_) and gradually generate ettringite (Aft). Calcite (CaCO_3_) and Na_2_CO_3_ will be generated after NaHCO_3_ infiltrates into concrete.

### 6.2. Microstructural Assessment

**Figure 9:**
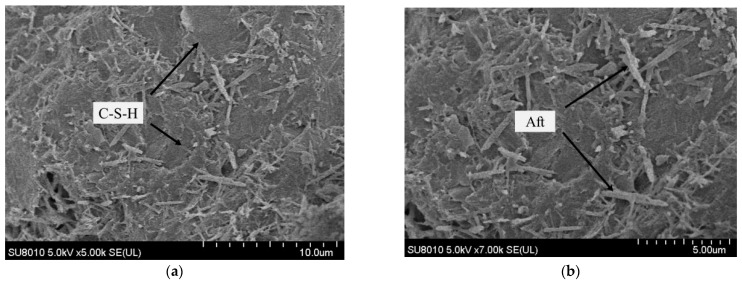
SEM images of concrete before FTCs. (**a**) 5000×. (**b**) 7000×.

**Figure 10:**
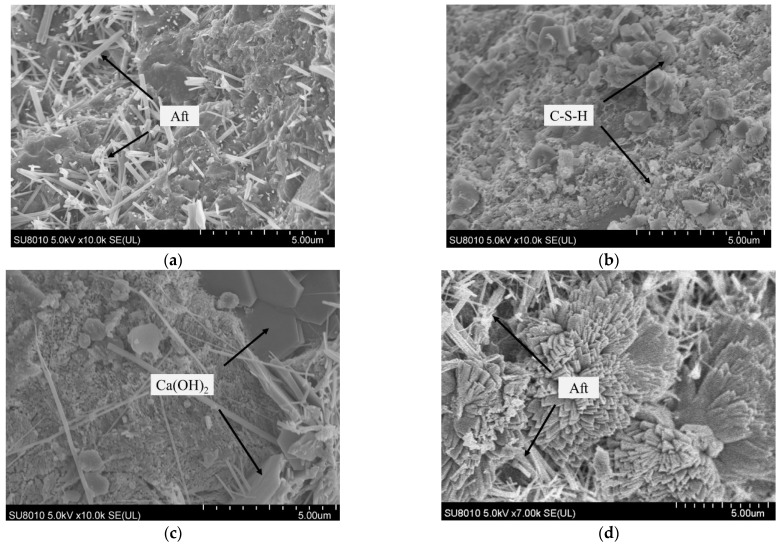
SEM image of concrete under freeze–thaw action in a (**a**) NaCl solution; (**b**) Na_2_SO_4_ solution; (**c**) NaHCO_3_ solution; and (**d**) composite salt solution.

The microstructural characteristics of concrete under FTCs in a salt solution are assessed here. The scanning electron microscopy images of the concrete structure near the erosion surface of the concrete specimen are shown in Figure 9 when concrete before FTCs action, and Figure 10 when the concrete experiences 100 FTCs in NaCl, NaHCO_3_, Na_2_SO_4_, and the composite solution.

The microstructure of concrete is dense without freeze–thaw cycling, and the microstructure of the concrete specimen changed after 100 FTCs in different salt solutions. The damage to the concrete caused by freeze–thaw action in a salt solution is more severe than that in freshwater [4]. The main components of cement concrete include the anchor sheet and flocculent C-S-H, needle-like ettringite (Aft), hexagonal plate-like Ca(OH)_2_, and rhombic hexahedral CaCO_3_. Although salt solutions will reduce the freezing point of the solution, the crystallization pressure of various salt solutions can severely damage the structure and lead to cracking. The freeze–thaw action will make the number of pores in the concrete structure gradually increase, the number of internal cracks increase, and make the structure of the concrete looser. The amount of overlapping needle-like ettringite within the concrete structure under freeze–thaw action in salt solutions increases significantly.

## 7. Correlation Analysis of Damage Performance

By comparing the variation rule of the compressive strength, DEM, and *T*_2_ spectrum of concrete under freeze–thaw action in different salt solutions, it can be seen that the damage value of concrete changes approximately linearly with the number of FTCs. The grey correlation degree method is applied to calculate the correlation degree and compare the relationship between the relevant performance change of concrete under different numbers of FTCs in different solutions [32]. Based on the change in the compressive strength of the concrete specimen, the relationship between the DEM and the *T*_2_ spectrum of the concrete specimen is calculated to study the correlation between each durability performance index of concrete. The closer the change rule between two indexes, the greater the grey correlation degree. The correlation coefficients are shown in Table 3.

It can be seen that the measured DEM of concrete is closely related to the compressive strength of concrete, and the change of each peak in the *T*_2_ spectrum of the concrete specimen is also highly correlated with changes in the compressive strength of concrete when it is subjected to FTCs in different salt solutions. The changes in each peak of the *T*_2_ spectrum are more similar to the changes in the mechanical properties of concrete specimens; when comparing the change in the total area of *T*_2_, and the correlation coefficients are both larger than 0.7. This shows that the measured DEM and the variation characteristics of the *T*_2_ spectrum of concrete specimens can both be used as indicators to evaluate the deterioration of the compressive strength of concrete under freeze–thaw action.

## 8. Conclusions

(1) Based on the rapid freeze–thaw experiments in the salt solution based on the characteristics of saline soil in western Jilin, China, it can be seen that the damage caused by FTCs is more severe in the composite salt solution than that in freshwater. By comparing the effects of FTCs in the three kinds of single salt solutions comprising the composite salt, the damage of the NaHCO_3_ solution to the compressive strength and DEM of concrete is more severe than that of the two other kinds of salt solutions, and the damage to the internal structure is more severe than the surface erosion of concrete under FTCs in a NaCl solution.
(2) When the concrete is subjected to freeze–thaw action in a salt solution, the moisture absorption of salt improves the pore saturation of concrete. The coupling effect of freshwater and crystallization pressure, which is generated when salt penetrates into the concrete, intensifies the damage to concrete. This damage makes the surface erosion and number of internal cracks gradually increase in the concrete; the increase in the middle pores and transition pores in the concrete is also more obvious.
(3) From the correlation coefficients of concrete under freeze–thaw action in freshwater and salt solutions calculated by the grey correlation degree, it can be seen that the change characteristics of the DEM and pore variation are similar to the compressive strength of concrete. The characteristics of the DEM and *T*_2_ spectrum can be used as non-destructive testing metrics to evaluate the compressive strength damage of concrete after FTCs.
(4) The durability research in this paper is mainly focused on the changes in the mechanical and physical properties of ordinary cement concrete blocks under rapid FTCs, and the performance variations in the air-entraining-agent-filled concrete, fibre-reinforced concrete, and ultra-high-strength concrete under the action of rapid FTCs in freshwater or salt solutions require further study. Further, the influence of the salt solution concentration on the related characteristics of concrete under rapid FTCs also needs to be further analysed.

## Figures and Tables

**Table 1:** Cement parameters.

Initial Setting Time (h)	Final Setting Time (h)	7-Day Flexural Strength (MPa)	7-Day Compressive Strength (MPa)	28-Day Flexural Strength (MPa)	28-Day Compressive Strengh (MPa)
2.85	5.3	7.5	42.2	7.6	56.7

**Table 2:** Proportioning of concrete (kg/m^3^).

Cement	Water	Coal Ash	Fine Aggregate	Coarse Aggregate	7-Day Compressive Strength (MPa)	28-Day Compressive Strength (MPa)
500	250	55	600	1105	32.8	42.5

**Table 3:** Correlation coefficients.

Solution	DEM	Total Area of *T*_2_	First Peak of *T*_2_	Second Peak of *T*_2_	Third Peak of *T*_2_
Freshwater	0.84	0.63	0.84	0.92	0.91
NaCl	0.90	0.63	0.96	0.83	0.90
NaHCO_3_	0.90	0.60	0.93	0.88	0.93
Na_2_SO_4_	0.98	0.65	0.98	0.97	0.98
Composite salt	0.78	0.59	0.83	0.73	0.79

## Data Availability

Not applicable.
